# Rethinking Colorectal Cancer Screening: Culturally Tailored Navigation for Mailed FIT Kits in 45- to 49-Year-Olds

**DOI:** 10.1007/s11606-025-10057-z

**Published:** 2025-12-08

**Authors:** David M. Callender, Lindsay Hauser, Cynthia Yoshida, Sarah Knight, Rebecca A. Krukowski, Melissa Warren, Taylor Krafchick, Keyri Lopez-Godoy, Ashley Banegas, Wendy Cohn

**Affiliations:** 1 Department of Medicine, University of Virginia School of Medicine, Charlottesville, VA USA; 2 Department of Public Health Sciences, University of Virginia School of Medicine, Charlottesville, VA USA; 3 University of Virginia School of Nursing, Charlottesville, VA USA

**Keywords:** Colorectal cancer (CRC) screening, Fecal immunochemical test (FIT), Patient navigation, Underserved populations

## Abstract

**Background:**

In response to rising colorectal cancer (CRC) incidence in younger adults, the US Preventive Services Task Force now recommends CRC screening to begin at age 45. Despite this change, screening rates remain low among 45- to 49-year-olds. Mailed fecal immunochemical test (FIT) programs with navigation have shown promise in increasing uptake.

**Objective:**

To evaluate the effectiveness of a mailed FIT program with culturally tailored navigation to improve CRC screening rates among adults aged 45–49 in a large, hospital-based safety-net primary care clinic.

**Methods:**

A quality improvement initiative identified eligible patients aged 45–49 not up to date with CRC screening. FIT kits were mailed following an invitation letter in English and Spanish with messaging based on the health belief model. Navigation was multi-modal including tailored patient education, bilingual phone calls, and reminder letters. Screening completion and follow-up outcomes were tracked via Epic and REDCap.

**Results:**

Of the 589 patients who received mailed FITs, 143 (24%) returned a completed test. Overall, Hispanic patients returned FITs at higher rates than non-Hispanic patients (29% vs. 21%), including both those who received navigation and those who did not. Navigation improved return rates, with 16% of outstanding FITs returned after outreach. Eleven tests were positive (8%), and 8 (73%) had a follow-up colonoscopy which identified 3 advanced adenomas.

**Conclusion:**

A culturally tailored mailed FIT intervention with navigation significantly improved screening rates among newly eligible adults, especially Hispanic patients. The findings highlight the importance of culturally tailored, multi-modal navigation to increase screening among younger, newly eligible adults and reduce screening disparities among underrepresented groups. Findings support broader implementation and suggest that navigation services can advance health equity in primary care. Future work should explore scalability and compare navigation to usual care.

## INTRODUCTION

In 2021, the United States (US) Preventive Services Task Force (USPSTF) and the US Multi-Society Task Force on Colorectal Cancer (CRC) screening lowered the recommended age for initiating average-risk CRC screening from 50 to 45.^1^ This change reflects the sharp rise in early-onset CRC incidence and mortality which now mirrors rates historically seen in older adults.^2,3^ While evidence demonstrates that initiating screening at age 45 is effective and cost-efficient, implementation of this guideline has lagged.^4–6^ Between 2019 and 2021, only 19.7% of eligible adults aged 45–49 were up to date with CRC screening.^7^

Colorectal cancer screening among newly eligible adults reveals disparities in implementation and uptake. Rates are especially low among uninsured individuals (7.6%), those with less educational attainment than a high school diploma (15.4%), and those identifying as Hispanic (16.1%).^7^ These patterns mirror long-standing inequities in CRC screening and may foreshadow persistent gaps in the newly eligible younger population.^8^ Stool-based testing, despite its effectiveness and lower resource burden, has been notably underutilized. Current screening efforts among 45- to 49-year-olds and underrepresented populations are often inadequate, largely due to barriers such as lack of awareness, accessibility, and other healthcare disparities.^9–12^ Screening rates were particularly low among 45- to 49-year-olds with only 2.4% reporting they were up to date with screening using stool-based testing.^7^ These patterns highlight persistent disparities and a widening gap in screening implementation.

Multiple barriers hinder the update of CRC screening in this younger cohort and in particular underrepresented groups. Limited awareness of the updated guidelines, lack of access to care, socioeconomic constraints, and structural health disparities all contribute to underuse.^9–12^ Early evidence suggests that these barriers may disproportionately affect younger adults, compounding existing inequities in preventive care.^11,12^ Additionally, resource constraints in primary care settings, such as provider availability and limited access to modalities like CT colonoscopy, further impede widespread adoption.^13^ Together, these factors hinder the timely and equitable adoption of early CRC screening.

Mailed fecal immunochemical test (FIT) programs with navigation have emerged as a promising solution to overcome these barriers. Trials in safety-net settings demonstrate that combining mailed FIT with patient navigation increases CRC screening completion, particularly among underserved populations, such as Hispanic communities.^14–16^ Navigation provides culturally tailored, personalized support that helps patients understand the importance of screening, overcome logistical challenges, and complete the testing process. For example, combining patient decision aids with navigation increased completion rates from 27 to 68% in an underserved patient population.^17^ Tailored programs have shown superior effectiveness compared with standard outreach.^18^

Yet evidence on the implementation of direct-to-patient FIT testing with navigation among adults newly eligible for screening (ages 45–49) remains limited. Understanding how these strategies perform in this targeted population is critical for achieving equitable uptake of the new guidelines.

Primary care settings can be central to addressing disparities by embedding navigation into routine screening. This paper presents results from a quality improvement study that evaluated how incorporating culturally tailored navigation, with the goal of improving uptake and equity among adults ages 45–49, particularly those from under-resourced populations.

## CONTEXT

We conducted this quality improvement study at a large, hospital-based primary care clinic (University Medical Associates (UMA)). UMA trains nearly 100 internal medicine residents and serves a diverse population of more than 10,000 patients. Hispanic patients account for 21% of the clinic population and 22% are Black. The safety-net environment and high proportion of uninsured patients shaped the design of our mailed FIT plus culturally tailored navigation intervention. Approximately 23% of patients lack health insurance, and 39% face significant barriers due to the distance they live from the clinic. In 2022, the clinic CRC screening rates were 54% for patients aged 45–75 and 26% for those aged 45–49 and defined as having any type of CRC screening completed within guideline-recommended time frames (e.g., colonoscopy within 10 years, FIT within 1 year). Among Hispanic patients, screening rates were lower, with 46% for ages 45–75 and just 21% for ages 45–49. When focusing on just FIT return rates prior to the intervention (12/2022 through 11/2023), 8% of UMA patients aged 45–49 with an open FIT order returned their test.

## PATIENT IDENTIFICATION

We included UMA patients aged 45–49 who had at least one office visit at UMA between 10/01/2020 and 10/01/2023, and were not “up-to-date” with CRC screening. Not “up-to-date” was defined as not having electronic health record (EHR) evidence of completing a fecal occult blood test in the past year, colonoscopy in the past 10 years, FIT/DNA in the past 3 years, CT colonography in the past 5 years, or flexible sigmoidoscopy in the past 5 years. Patients who had not had any actual clinic encounter (e.g., research visits, COVID testing, vaccination alone); who had CRC cancer or a history of colectomy; or who were receiving hospice care, were living in long-term care institutions, and had frailty and advanced illness or had dementia; or who were actively receiving palliative care were excluded. We identified eligible patients through an Epic (Version Aug2023; Epic Systems Corporation, Madison, WI) query which generated a report of all eligible patients aged 45–49 without evidence of CRC screening and based on the criteria outlined above. The Epic query identified patient race, ethnicity, gender, MyChart activation status, FIT orders, colonoscopy orders, and results. Data were linked to navigation records using medical record numbers and stored in a secure REDCap database (Version 14.0; Vanderbilt University, Nashville, TN).

## INTERVENTION

### Project Description

This project was categorized as quality improvement by the University of Virginia Institutional Review Board and utilizes the SQUIRE 2.0 framework for quality improvement reporting. It included direct-to-patient mailing of FIT kits with culturally tailored navigation support for adults aged 45–49 who were not up to date with colorectal cancer screening. The goal of the intervention was to improve from the baseline UMA clinic FIT return rate of 8%. The intervention involved three steps: (1) culturally tailored educational outreach and FIT mailing, (2) navigator phone call, (3) follow-up reminder letter.

### Step 1: Culturally Tailored Educational Outreach and FIT Mailing

**Figure 1.**
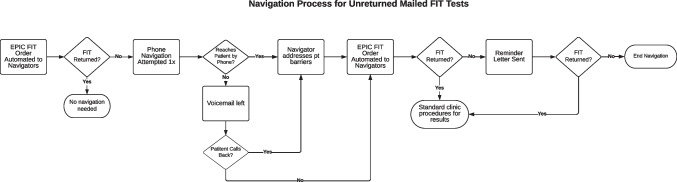
Navigation workflow of unreturned FITs.

**Figure 2.**
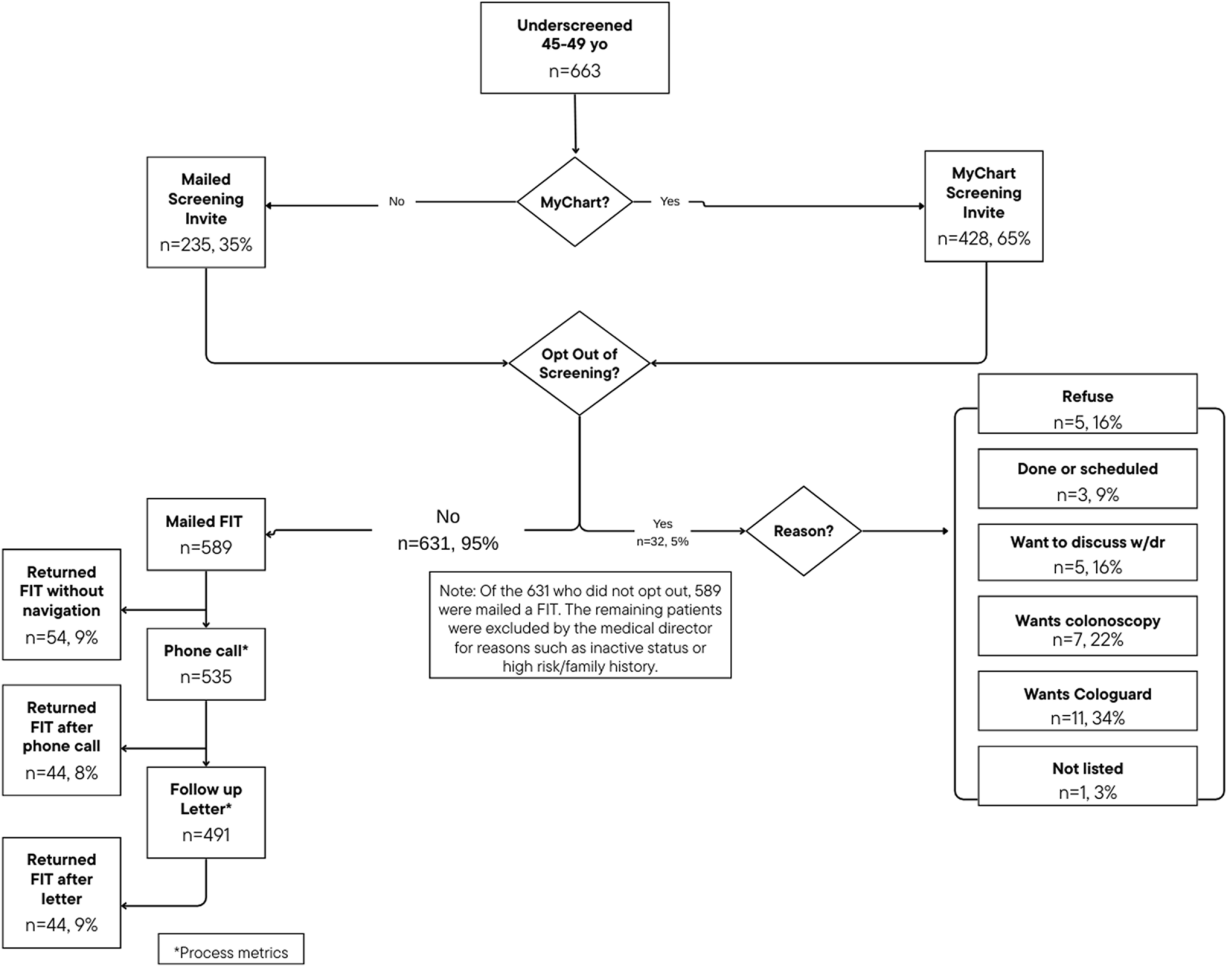
Mailed FIT quality improvement study workflow.

The quality improvement workflow (Fig. 1) details the process used to segment patients based on their patient portal activation status. Patients with an active MyChart account in the EMR received digital educational outreach through the portal. Those without an active MyChart account were assigned to receive mail-based educational outreach. The workflow also included opt-out procedures, test distribution, and follow-up procedures to ensure comprehensive coordination throughout the project. We used the health belief model (HBM) to develop materials inviting patients to complete colorectal cancer screening. The invitation informed patients they were eligible to be screened and addressed the key HBM constructs (Table 1). An infographic meeting the needs of individuals with low health literacy was also included in the invitation which provided details on all CRC screening options (Fig. 2). All communication and accompanying materials were provided in English and Spanish. We informed patients (via a portal message or mailing as previously mentioned) they would be receiving a screening test in the mail in approximately 1 week and were given the opportunity to opt out by using a QR code or electronic form. Patients who opted out were asked to select a reason from the following options: already completed screening, preferred to discuss CRC screening with their provider, preferred colonoscopy or Cologuard, or did not wish to be screened at this time.

**Table 1:** Invitation to Screen Health Belief Constructs

Health belief construct	Screening invitation language
Perceived susceptibility	In fact, about 1 in 20 people will have CRC in their lifetime
Perceived severity	CRC is the second leading cause of cancer deaths
Perceived benefits	CRC can be prevented, or treated, if found early
Perceived barriers	We will mail you a FIT test which you can do at home and mail back

One week after the initial invitation, the clinic’s medical director placed FIT orders for all patients who had not opted out, and we mailed the FIT kits. FIT packages included an educational postcard explaining the purpose of the FIT, an infographic with instructions for completion, a pre-labeled FIT, and a pre-paid postage return mailer. Additionally, uninsured patients had their lab fees waived through the inclusion of a pink slip in their envelopes, ensuring cost-free processing and addressing potential financial barriers.

### Step 2: Navigator Phone Call

**Figure 3.**
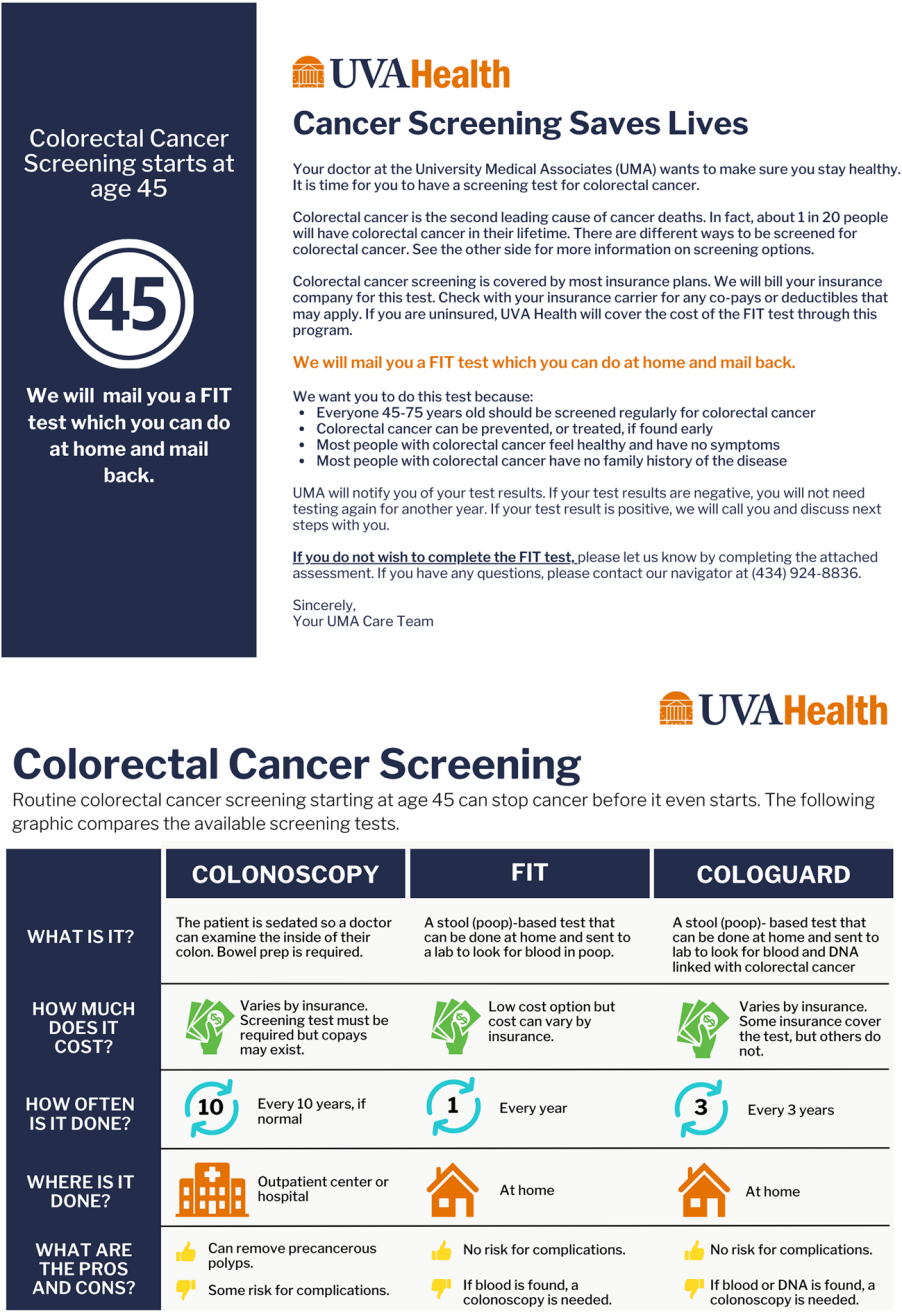
CRC screening invitation.

We used the REDCap database to track and document FIT returns. Patients who did not return their FIT after 10 days were eligible for navigation. The navigation workflow for patients with unreturned FITs is shown in Fig. 3. Four trained navigation specialists provided navigation which included one clinic nurse who also provided escalation oversight when necessary. All Spanish-speaking patients, as identified by their primary language in the EMR, were navigated by a bilingual navigator (KLG), allowing for language-concordant and culturally aligned communication. The navigators attempted one phone call with patients. If a patient did not answer, a scripted voicemail was left reminding them to complete their FIT test and provided a call back number. When patients were able to be reached by phone, the navigator used a culturally adapted script to identify and address common barriers to FIT return. Barriers addressed included forgetting to complete the test, questions about how to complete the test, concerns about returning the test, lost or misplaced test, fear of test results, embarrassment about the test, uncertainty about the purpose of the test, concerns about effectiveness, and other.

### Step 3: Follow-Up Reminder Letter

Any patient with an outstanding FIT after 2 weeks of the initial navigation attempt was mailed a final reminder letter encouraging them to complete their test.

## MEASURES

Our primary outcome was FIT return rate among eligible patients. Our secondary outcome was FIT return rates by ethnicity. Other process metrics included the number of patients reached by portal or mail, opt-out number and reasons, number who were mailed the FIT, number who received a phone call, and the number who received a follow-up letter.

## ANALYSIS

FIT return rate was assessed by identifying the proportion of patients who returned their mailed FIT kit. To understand the impact of different types of navigation on FIT return rates, we calculated the proportion of patients who returned their FIT kit following the implementation of each navigation step. The chi-square test of independence was used to test for statistically significant differences between Hispanic and non-Hispanic patients who returned their FIT after the introduction letter in step 1, those who returned after the phone call in step 2 of navigation and then those who returned after the follow-up letter in step 3 (Table 2). The chi-square test of independence was also used to test for differences in return rate by sex and race. We performed statistical analyses using SPSS Software (Version 29.0.2.0; International Business Machines Corporation, Armonk, NY).

**Table 2:** Cohort Description

Method of initial contact	All ethnicities		Hispanic		Non-Hispanic	
	*N*	Percent	*N*	Percent	*N*	Percent
MyChart	428	65%	109	43%	319	78%
Mail	235	35%	147	57%	88	22%
Sex						
Male	290	44%	87	34%	203	50%
Female	344	52%	156	61%	188	46%
Unknown	29	4%	13	5%	16	4%
Race						
African American	130	20%	4	2%	126	31%
White	295	44%	72	28%	223	55%
Other	238	36%	180	70%	58	14%

## RESULTS

The quality study took place between December 4, 2023, and April 1, 2024. Figure 2 outlines the process and metrics associated with each step. Of the 589 patients who were mailed the FIT kits, 142 (24%) returned (Table 3). Overall, Hispanic patients returned FITs at a significantly higher rate than patients who are not Hispanic (28.8% and 21.2% respectively). Within individual navigation steps, no statistically significant differences in return rates were observed by ethnicity.

Each navigation step did bring in additional FIT returns. Following the phone call in step 2, an additional 8% (*n* = 44) of patients returned their FIT. Among the 491 patients who still had not returned their FIT 2 weeks after the initial call, a final reminder letter was mailed. This final mailing led to an additional 9% (*n* = 44) of patients returning their FIT. This left 447 unreturned mailed FITs.

**Table 3:** FIT Return Rates

		**Total # eligible**	**Fit returned, yes (%)**	**Fit returned, no (%)**	***χ***^**2**^ ***p*-value**
**Overall**	Needs screening	589	142 (24.1)	447 (75.9)	
	Ethnicity				
	Hispanic	226	65 (28.8)	161 (71.2)	0.040*
	Not Hispanic	363	77 (21.2)	286 (78.8)	
**Stage 1: invitation letter**	Needs screening	589	54 (9.2)	535 (90.8)	
	Ethnicity				
	Hispanic	226	29 (12.8)	197 (87.2)	0.099
	Not Hispanic	363	25 (6.9)	363 (93.1)	
**Stage 2: navigation call**	Needs screening	535	44 (8.2)	491 (91.8)	
	Ethnicity				
	Hispanic	197	17 (8.6)	180 (91.4)	0.551
	Not Hispanic	338	27 (8.0)	311 (92.0)	
**Stage 3: final letter**	Needs screening	491	44 (9.0%)	447 (91.0)	
	Ethnicity				
	Hispanic	178	19 (10.7)	159 (89.3)	0.555
	Not Hispanic	311	25 (8.0)	286 (92.0)	

*p-value < 0.05

Overall FIT return rates did not differ by sex. They did, however, differ significantly by race, with African American (12%) patients returning at lower rates than White (27%) or other (26%) patients (*p*-value = 0.030).

Of the 142 total returned FITs, 8% (*n* = 11) were positive. Within 3 months, 73% (*n* = 8) of patients with positive FITs had completed colonoscopies. Of those colonoscopies, 38% (*n* = 3) identified advanced adenomas. The three colonoscopies that were not completed were due to scheduling and preparation barriers.

## DISCUSSION

The results of this quality improvement project underscore the significant impact that multi-modal navigation services can have to improve FIT return rates in patients aged 45–49, particularly among Hispanic populations. Overall, the navigation and mailed FIT campaign produced higher FIT return rates (ADD %) than at baseline in the UMA clinic (ADD %). The higher return rates among Hispanic patients (29% compared to 21% for non-Hispanics), even solely in response to the initial invitation letter and mailed FIT (no navigation; 13% compared to 7% for non-Hispanics), may indicate that our tailored approach, including the provision of Spanish-language materials and home testing kits, could have played a role in enhancing engagement and adherence to screening guidelines. It is also noteworthy that Hispanic patients more often needed the invitation letter by mail versus digitally. Forty-three percent of Hispanic patients used MyChart compared to 78% among non-Hispanic patients. This difference indicates that relying solely on digital communication platforms may inadvertently exclude certain populations from receiving timely health information. This underscores the necessity of using multiple communication channels to ensure equitable access to healthcare services, especially for populations with limited digital access.

Of the 142 returned FITS, 8% were positive with 10% among non-Hispanic individuals and 5% among Hispanic individuals. Among those with a positive FIT result, 73% completed a follow-up colonoscopy within 3 months. The overall FIT positivity rate is slightly higher overall but similar to the findings in a large review of ten studies involving 664,159 FITs that found a 4.9% positivity rate among individuals aged 40–49 years.^19^ In addition, a large retrospective cohort study conducted by Kaiser Permanente in Northern California, Washington, and Colorado reported a 3.6% FIT positivity rate and a 64.9% colonoscopy completion rate within 3 months of a positive result, which is also similar to our findings.^20^

One of the key findings compared to the literature was our higher completion rate of colonoscopies following a positive FIT among all groups. A recently published systematic review found that to improve follow-up colonoscopy rates after abnormal FIT results, multilevel interventions are crucial. Clinics with higher completion rates used patient registries, assigned communication responsibilities to at least two team members (including a nurse or medical assistant), and implemented consistent follow-up processes.^21^ Our navigation utilized similar strategies integrated into broader interventions addressing barriers such as transportation, navigation, and financial assistance. Specifically, navigators engaged patients in discussions to identify feasible solutions to transportation challenges, including transportation vouchers provided through social work, cost-protective public transportation options, and culturally sensitive alternatives such as community carpools. By tackling patient, clinic, and system-level obstacles, this culturally tailored navigation can enhance care quality and advance health equity, particularly in safety-net settings, addressing social determinants of health and reducing disparities in CRC care.

This project was implemented with the intention of improving CRC screening rates within UMA’s clinic. The improved results show potential to more rigorously design and evaluate as a research study to better test the impact of navigation components on FIT return rates.

### Limitations

Our study has several limitations. Our small sample size limits meaningful comparison to larger cohorts. Any conclusions drawn from our observed higher positivity rates (5% among Hispanic and 10% among non-Hispanic patients) relative to the 3.6% rate reported in a large observational study of the same age group may not be comparable.^20^ However, our findings were more aligned with screening rates observed in the first year after lowering the age for CRC screening in this age group (4.5%).^22^ Additionally, a systematic review and meta-analysis of similar-aged individuals reported a positive rate of 4.9%.^21^ The most comparable results were seen in a mailed FIT outreach study conducted in a 45–49-year-old community health center population, which reported a 6% positive rate.^23^

Our findings may be attributed to variations in population risk factors and the fact that our quality improvement study was not a retrospective cohort study. To more accurately assess the effectiveness of the culturally tailored navigation intervention, future studies should include randomization and a control group receiving usual care. Additionally, we did not systematically quantify balancing measures (e.g., navigator time, cost impact), limiting full understanding of implementation burden. We did collect information quarterly from clinicians who reported that the handoff of information was excellent and that patients were more readily able to complete screening.

### Future Directions

This culturally tailored navigation intervention is feasible in safety-net settings, but sustainability requires dedicated resources. Navigation was supported by grant funding; embedding this role into clinic workflows and securing institutional support will be essential for long-term continuation. Scaling up may involve automating EMR queries, developing bilingual digital outreach, and hiring dedicated CRC screening leads. Future research should compare culturally tailored navigation to usual care in randomized designs, evaluate cost-effectiveness, and test adaptation across diverse health systems.

## CONCLUSION

In conclusion, the integration of culturally tailored navigation services within primary care settings holds significant promise for improving cancer screening adherence and reducing disparities, particularly among underrepresented populations such as Hispanics. By continuing to refine and expand these services, we can work towards more equitable healthcare outcomes for all individuals.

## Data Availability

Data are available by request to the corresponding author.
